# Fc receptors and the diversity of antibody responses to HIV infection and vaccination

**DOI:** 10.1038/s41435-022-00175-7

**Published:** 2022-06-10

**Authors:** Li-Yun Lin, Raphael Carapito, Bin Su, Christiane Moog

**Affiliations:** 1 Laboratoire d’ImmunoRhumatologie Moléculaire, Institut national de la santé et de la recherche médicale (INSERM) UMR_S 1109, Institut thématique interdisciplinaire (ITI) de Médecine de Précision de Strasbourg, Transplantex NG, Faculté de Médecine, Fédération Hospitalo-Universitaire OMICARE, Fédération de Médecine Translationnelle de Strasbourg (FMTS), Université de Strasbourg, Strasbourg, France; 2 Laboratoire d’Immunologie, Plateau Technique de Biologie, Pôle de Biologie, Nouvel Hôpital Civil, Strasbourg, France; 3 Beijing Key Laboratory for HIV/AIDS Research, Sino-French Joint Laboratory for Research on Humoral Immune Response to HIV Infection, Clinical and Research Center for Infectious Diseases, Beijing Youan Hospital, Capital Medical University, Beijing, China; 4 Vaccine Research Institute (VRI), Créteil, France

**Keywords:** HIV infections, Genetic predisposition to disease, Infection

## Abstract

The development of an effective vaccine against HIV is desperately needed. The successive failures of HIV vaccine efficacy trials in recent decades have shown the difficulty of inducing an appropriate protective immune response to fight HIV. Different correlates of antibody parameters associated with a decreased risk of HIV-1 acquisition have been identified. However, these parameters are difficult to reproduce and improve, possibly because they have an intricate and combined action. Here, we describe the numerous antibody (Ab) functions associated with HIV-1 protection and report the interrelated parameters regulating their complex functions. Indeed, besides neutralizing and Fc-mediated activity, additional factors such as Ab type, concentration and kinetics of induction, and Fc-receptor expression and binding capacity also influence the protective effect conferred by Abs. As these parameters were described to be associated with ethnicity, age and sex, these additional factors must be considered for the development of an effective immune response. Therefore, future vaccine designs need to consider these multifaceted Ab functions together with the demographic attributes of the patient populations.

## Introduction

According to World Health Organization (WHO) data from 2020, 37.7 million people are living with HIV-1/AIDS and 68% of them are Africans [1]. In contrast to western Europe and America, where subtype B is predominant, subtype A is largely distributed in Eastern Europe and Central Asia and subtype C in East Asia. Africa shows the highest HIV-1 diversity with subtypes A and D in eastern Africa, C in southern Africa, A, G, CRF02_AG, and CRF06_cpx in western Africa, and B and CRF02_AG in northern Africa [2–4]. To fight against and end the HIV-1 pandemic, an efficient protective vaccine is needed. However, due to the high diversity of HIV-1 subtypes, vaccines need to induce antibodies (Abs) with broad inhibitory activity, i.e., antibodies able to inhibit numerous HIV-1 variants. This requirement is considered as one of the main limitations for the development of an efficient HIV vaccine [5, 6].

Over more than three decades, several HIV-1 vaccine trials have been conducted all over the world [7]. However, in HIV-1 vaccine history, only the RV144 phase III trial performed in Thailand showed a statistically significant decreased risk for HIV-1 acquisition at 42 months (31.2%) [8]. Interestingly, analysis of immune correlates for risk showed that Abs binding to the V1V2 region of gp120 correlated with a decreased risk for infection [9]. The IgG1 and IgG3 subclasses mediating antibody-dependent cell-mediated cytotoxicity (ADCC) seem to play a predominant role in protection against HIV-1 acquisition [10]. Moreover, the concentration of plasma envelope (Env)-specific IgA Abs was found to be directly correlated with a higher risk for HIV acquisition [10, 11]. These correlates of risk highlight the predominant role of isotypes and Fc-mediated functions in addition to the previously known protective role of neutralizing antibodies (NAbs). Knowledge of these new factors opens windows of opportunities for innovations in inducing a broad inhibitory humoral immune response to fight HIV and introduces new parameters to be considered, such as Fc domain/Fc receptor (FcR) interactions [12–17].

## Antibodies and the pleiotropic function of the humoral response

### Induction of HIV-specific Abs of various isotypes

**Fig. 1:**
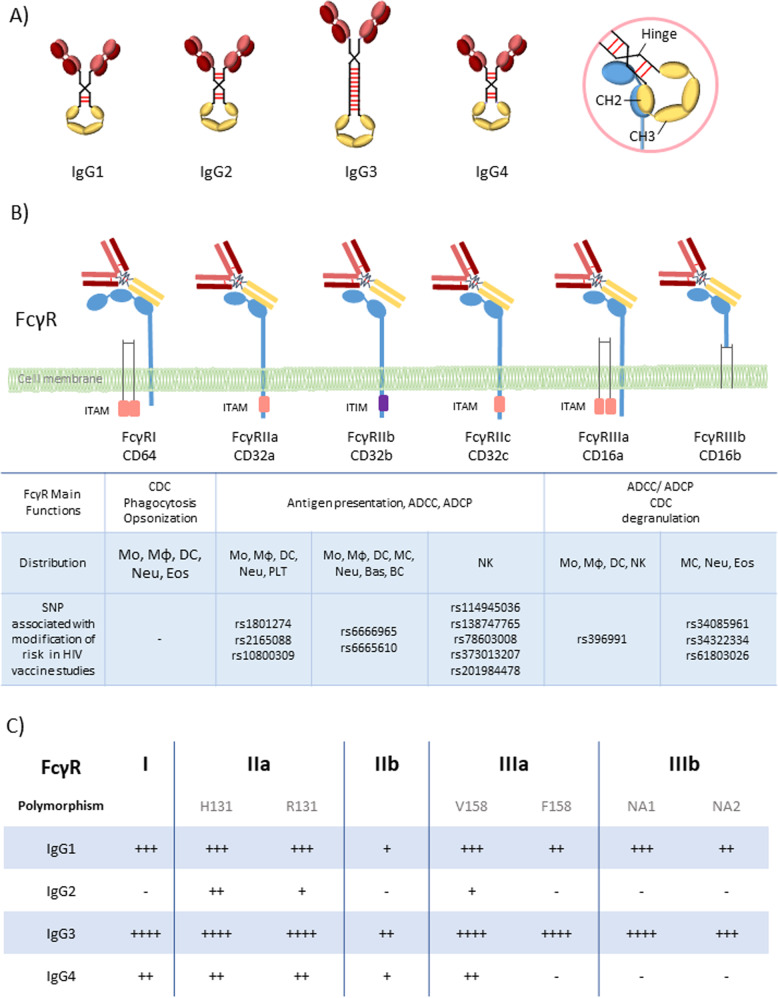
Antibodies and FcR-mediated functions. **A** IgG subclasses. **B** Fc gamma receptors (FcγRI, FcγRIIa, FcγRIIb, FcγRIIc, FcγRIIIa, FcγRIIIb), their main function, polymorphisms, and distribution on immune cells. **C** FcγR binding affinities of IgG subclasses. CDC complement dependent cytotoxicity, ADCC antibody-dependent cellular cytotoxicity, ADCP antibody-dependent cellular phagocytosis, Mo Monocyte, Mϕ Macrophage, DC Dendritic cell, MC Mast cell, Neu Neutrophil, Bas Basophil, Eos Eosinophil, NK Natural killer cell, BC B cell, PLT Platelet.

The B cells of the immune system produce Abs that are classified into five major immunoglobulin (Ig) classes or isotypes: IgM, IgG, IgA, IgD, and IgE [18]. IgG is further divided into four subclasses (Fig. 1A) that are diversely distributed according to ethnicity, sex and age, with IgG1, IgG2, IgG3, and IgG4 representing 60–72%, 20–31%, 5–10%, and <4% of total IgG, respectively [19]. IgG subclass prevalence has been reported to change over time following the course of disease and symptoms [20]. Following HIV-1 infection, the adaptive immune response predominantly induces IgG1, IgG3 and IgA [21]. In the RV144 vaccine trial, high levels of HIV-1-specific IgG3 and low Env-specific IgA correlated with a decreased risk of HIV-1 infection [10]. The various Ab isotypes and subclasses bind differently to Fc receptors at the surface of immune cells, including dendritic cells and mainly macrophages (Fig. 1B). As these cells are the best-in-class antigen-presenting cells, different Ab isotypes and subclasses directly affect Ab binding to antigen-presenting cells, modulating immune cell activation and consequently the quality of the humoral immune response that is induced [22]. Comprehensively interrogating the extensive biological Ig diversity in patients may provide critical insights that can guide the development of effective Ab-based vaccines and therapies.

### Two main antibody functions observed in HIV-infected patients and in vaccine trials: neutralization and Fc-mediated functions

NAbs protect cells from pathogens or infectious particles by inhibiting any effect leading to infection via the binding of their Fab domain to the infectious agent (Fig. 1B) [23, 24]. Studies of the passive injection of broadly NAbs in nonhuman primate (NHP) models demonstrate their high potential for conferring protection against HIV acquisition [23, 25]. Considering these data, immunogens aiming to induce the production of these NAbs were developed [23, 26]. Many vaccines have been designed to induce Abs targeting the envelope glycoproteins of the virus, mainly gp120 or gp160 [26–28]. However, these vaccines failed to induce broadly NAbs. Indeed, the production of broadly NAbs is extremely difficult to induce due to the need for an extensive maturation process [29, 30].

**Fig. 2:**
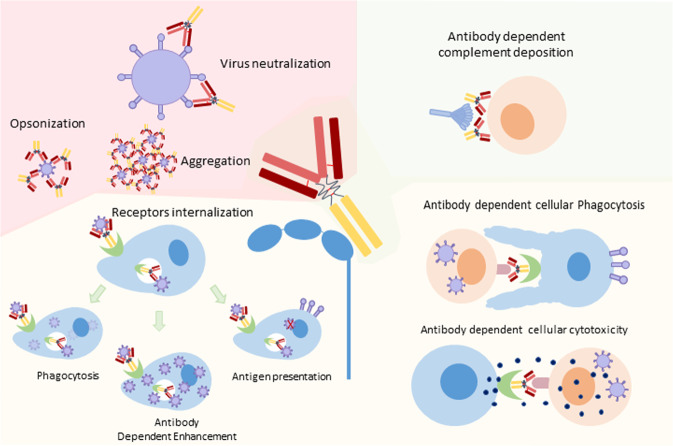
HIV antibody functions. The functions are dependent on different Ab domains: The Fab domain is involved in virus neutralization, opsonization and aggregation; the Fc domain of Ab induces the activation of the complement system; dual binding of Ab via Fab and Fc domains leads to Fc-mediated antibody function: antibody-dependent cellular phagocytosis and antibody-dependent cellular cytotoxicity; FcR internalization may lead to phagocytosis, antigen presentation or antibody-dependent enhancement.

The success of the RV144 vaccine trial supported the development of new vaccine designs for the induction of Abs with additional functions, mainly Fc-mediated Ab functions [31, 32]. It has been proposed that several Fc-mediated mechanisms, including ADCC, antibody-dependent cellular phagocytosis (ADCP), antibody-dependent complement deposition (ADCD), aggregation and immune activation, participate in HIV inhibition (Figs. 1B, 2) [14, 33–37]. In addition, viruses can be directly opsonized by phagocytosis *via* Ab and FcR binding. The virus is then destroyed, and digested peptides can be retrieved by antigen-presenting cells for T cell activation (Fig. 2) [17, 34, 38, 39]. If the virus escapes this lysis process, opsonized virus entry may also lead to increased infection by a process called antibody-dependent enhancement (ADE) [40]. This ADE function should of course be avoided [41–43]. All these different Fc-mediated mechanisms involve the binding of the Fc domain of the Ab to the Fc receptor present on immune cells. The Fc-mediated functions of Abs are therefore also directly interconnected with FcR expression at the surface of immune cells [44, 45].

## Modulating FcR expression at the surface of immune cells

FcRs are cell surface glycoproteins that bind to the Fc domain of Abs. This binding varies according to the isotype and subclass of the Ab but also according to the type of FcR (Fig. 1B, C) [44–46]. These FcRs are differentially expressed on most immune cells, including natural killer (NK) cells, monocytes, macrophages, eosinophils, dendritic cells, B cells and even some T cells [17, 46]. There are three family classes of FcRs (I, II, and III), each of which comprises a different number of proteins: FcγRI, FcγRIIa, FcγRIIb, FcγRIIc, FcγRIIIa and FcγRIIIb (Fig. 1B) [18]. All human FcγRs except FcγRIIB signal through an immunoreceptor tyrosine-based activating motif (ITAM), whereas FcγRIIB delivers inhibitory signals through an immunoreceptor tyrosine-based inhibitory motif (ITIM) [4, 46]. The diversity of human FcγRII and III is further increased by single nucleotide polymorphisms (SNPs) in their extracellular domains, the most studied of which are H131R in FcγR gene FCGR2A, 126C>T in FCGR2C, F158V in FCGR3A, and NA1/2 in FCGR3B (Fig. 1C). FcγRIIC has an unusual structure and is generated by an unequal crossover between FcγRIIA and FcγRIIB. FCGR2C signals through the ITAM similarly to FCGR2A. FcγRIIC (126C>T), rs114945036 presumably lead to an open reading frame with an atypical FcR protein sequence.

Importantly, the different FcR polymorphisms of the host need to be considered when analyzing FcR-mediated functions of Abs. FcγR SNPs will impact both on the the binding to the complementary Fc portion of the Abs and on the expression or activation state of the cells [46] (Fig. 1B). Increasing evidence suggests that FcγR SNPs impair receptor expression on DCs, which in turn influences the risk for HIV infection and vaccine efficacy [15, 16, 47]. Interestingly, a combination of polymorphisms may also influence FcR expression, such as the combination of rs1801274 and rs10800309 in the FcγRII coding gene FCGR2A, which affects the expression level of FcR on immature dendritic cells [48]. FcγRIIIA polymorphism appears to modify NK cell activation and, as a consequence, ADCC activity [49]. Specific polymorphisms at the FCGR2A (encoding Arg or His at position 131) and FCGR3A (encoding Phe or Val at position 158) gene loci have been associated with an HIV vaccine benefit [50]. The rs396991 SNP leads to an increased binding capacity of Abs for FcγRIIIA, which is the main receptor involved in ADCC, suggesting that the vaccine efficacy may be related to an increased efficacy of this function. More recently, Li et al. described that a tag SNP (rs114945036) in FCGR2C (126C>T, presumably leading to a stop codon or an open reading frame) was significantly associated with protection against infection with a subtype AE HIV-1 strain in the RV144 vaccine clinical trial [51]. The direct effect of this SNP is not well documented. Authors propose that it may lead to an alternative splicing, bypassing the FCGR2C-Stop codon to encode a product with an atypical FcR protein sequence, thereby modifying FcR expression or accessibility on cells [51].

Overall, the interplay between IgG subclasses, multiple FcRs and polymorphisms thereof contribute to the complexity of the Fc-mediated response [15, 46]. As a consequence, numerous studies have analyzed the association between FcR genes or their polymorphisms and the evolution of HIV disease or vaccine protection (Table 1) [50–55].

**Table 1:** HIV vaccine trials.

Vaccine trial	Year	Location	Target population	Vaccine	Ab function		Fc receptor			Vaccine efficacy	Ref.
					Neutralization	Fc mediated	FcR	Polymorphism	Association with risk of infection		
VaxSyn	1987	Canada	72 adults	Recombinant envelope glycoprotein subunit (rgp160) of HIV	Low Tier 1	*NFD*	–	–	–	No	[66, 67]
HIVAC-1e	1988	USA	35 male adults	Recombinant vaccinia virus designed to express HIV gp160	N/F	ADE	–	–	–	No	[68, 69]
Vax004	1998–2002	North America	5417 MSM and 300 women	AIDSVAX B/B gp120 with alum	Tier 1	ADCC ADCP	FCGR2A	rs1801274	↓	No	[37, 50, 52, 77, 84]
							FCGR3A	rs397991	↑		
Vax003	1999–2003	Thailand	2545 mem and women IDUs	AIDSVAX B/E gp120 with alum	Tier 1	ADCC	–	–	–	No	[77, 83, 84]
STEP/HVTN502	2004–2007	North America, Caribbean South America, and Australia	3000 MSM and heterosexual men and women	MRKAd5 HIV-1 gag/pol/nef trivalent vaccine	Low Tier 1	*NFD*	–	–	–	1.4 increased risk of infection	[70, 71, 74, 75]
Phambili/HVTN503	2003–2007	South Africa	801 adults	rAd5 (gag/pol/nef)	Low Tier 1	*NFD*	–	–	–	1.7 increased risk of infection	[72, 73]
RV144	2003–2009	Thailand	16,402 community-risk men and women	ALVAC-HIV (vCP1521) and AIDSVAX B/E vaccine	Low Tier 1	ADCC ADCP	FCGR2C	rs114945036 rs138747765 rs78603008	↓	31.2% decreased risk of infection	[8, 10, 51, 54, 82, 83, 85, 86]
HVTN505	2009–2013	USA	2504 men or transgender women who have sex with men	Three vaccinations with DNA encoding HIV clade B gag, pol and nef as well as env from HIV clades A, B and C followed by an Ad5 vector-based vaccine encoding clade B gag and pol as well as env from clades A, B and C	Low Tier 1	ADCC ADCP	FCGR2A	rs2165088	↓	No	[54, 81, 87]
							FCGR2C	rs138747765 rs78603008 rs373013207 rs201984478	↑		
							FcGR3B	rs34085961 rs34322334 rs61803026	↑		
							FCGR2B	rs6666965 rs6665610	↓		
HVTN305	2012–2017	Thailand	162 women and men	ALVAC-HIV and AIDSVAX B/E vaccine	Low Tier 1	ADCC ADCP	–	–	–	No	[86, 88]
HVTN306	2013–2020	Thailand	360 men and women aged 20–40 years old	ALVAC-HIV and AIDSVAX B/E vaccine	Low Tier 1	ADCC ADCP	–	–	–	No	[89, 90]
HVTN097	2012–2013	South Africa	100 black Africans (men and women) aged 18–40 years old	ALVAC-HIV (vCP1521) and AIDSVAX B/E vaccine	Low Tier 1	ADCC ADCP	–	–	–	No	[91]
HVTN100	2015–2018	South Africa	252 men and women	ALVAC-HIV (vCP2438) and bivalent subtype C gp120/MF59	Low Tier 1	ADCC ADCP	–	–	–	No	[92–94]
HVTN705/Imbokodo	2017–2021	Sub-Saharan Africa	2637 women ages 18 to 35 years	Ad26.Mos4.HIV, adjuvanted clade C and Mosaic gp140 HIV bivalent vaccine	–	–	–	–	–	Comparing with RV144, unable to improve the efficacy on Sub-Saharan Africa women	[31, 76, 78–80]

Illustration of completed and documented or on-going major phase 1b to phase 3 HIV trials that analyzed the Ab and/or Fc Receptor functions.

*NFD* no Fc-mediated function detected, *–* no related publications found.

## Effect of ethnicity, sex, and age on Fc-mediated Ab response to HIV

Several studies have shown that serum Ig concentrations vary according to ethnicity, sex, and age. Total IgG and IgA levels increase with age and reach the adult concentration at ~10 years of age. Thereafter, the levels of serum IgG were found to be significantly reduced with age, and the level of IgA was found to be maintained. Total IgG and IgA concentrations are higher in Black populations than in White populations [19, 56, 57]. A similar result of higher total IgG levels in HIV-infected Africans than in Caucasians and Hispanics was also found [57–60]. Notably, all these studies comparing Ab profiles according to ethnicity were performed in individuals living in the same country. The difference in Ab responses in Africans living in Africa and Caucasians living in Europe or the USA needs to be investigated to integrate the effect of geographic origin in these studies.

In addition, age-related differences in clonal expansion with decreased IgA levels and skew toward IgG2 were observed after influenza vaccination [61, 62].

These results illustrate the importance of Ab classes in vaccine studies. This difference in Ab isotypes and concentrations according to ethnicity, age and sex may directly impact FcR functions and influence the efficacy of Ab induction in HIV-vaccinated individuals.

The demonstration of the role of Fc-mediated function also brings into question the importance of FcR features. The frequencies of SNPs of FcR genes differ significantly between ethnic groups [63–65]. These differences may strongly modify the association found between FcR polymorphisms and HIV-1 protection or disease outcome. In Kawasaki disease for example, the association with the FCGR2C-ORF haplotype becomes evident only when Asians, in whom FCGR2C-ORF is a nearly absent haplotype, are excluded from the cohort [64].

Overall, analyzing Fc-mediated Ab functions without considering ethnicity, sex, and age is hazardous. These factors need to be considered for genotype/phenotype association studies, as well as for the analysis of FcR involvement in HIV vaccine trials.

## FcR and Ab functions in vaccine trials

During the past three decades, several HIV-1 vaccine trials have been performed all over the world. The first vaccine trial tested the recombinant envelope glycoprotein subunit (rgp160) in 72 adults. This vaccine showed induction of NAbs but not Fc-mediated Ab responses [66, 67]. The second HIV-1 trial (HIVAC-1e) used recombinant vaccinia virus that expressed HIV-1 gp160, and its administration resulted in no induction of neutralizing Ab or Fc-mediated Ab responses, even though ADE was detected [68, 69]. Whether this lack of detectable Ab function was due to technical issues needs to be further assessed. Thereafter the following vaccine trials using envelop antigens succeeded in inducing both neutralizing and Fc-medicated Ab responses (Table 1). Of note, the CD4^+^ T cell-driven HIV immunogens used in the HVTN502 and HVTN503 vaccine trials did not contain envelop antigens, and led to an increased risk of infection [70–75]. FcR variants and their potential association with a decreased risk for infection were further investigated in three vaccine trials: Vax004, HVTN505 and RV144 (Fig. 1B). Although the Vax004 and HVTN505 vaccine strategies did not show efficacy, distinct FCGR polymorphisms have been associated with either an increased or decreased risk for HIV-1 acquisition (Table 1). For the RV144 vaccine trial conducted in Thailand, an association between the FCGR2C rs114940536, rs138747765, rs78603008 polymorphisms and a decreased risk for HIV acquisition was shown [51]. While focusing on fighting the HIV-1 pandemic in Africa, a similar strategy to that used in the RV144 trial was initiated in the South African area [76–79]. This trial, called HVTN702, did not reach the efficacy requirement of RV144 and was therefore stopped prematurely [80]. This failure could be explained by the fact that Black South Africans do not possess the FCGR2C haplotype that was associated with increased vaccine efficacy in the RV144 trial [63]. Collectively, the differences in FCGR2C polymorphisms in South Africa versus Thailand highlight the need for further mechanistic investigations to define the functional relevance of FcR polymorphisms in HIV-1 protection, especially in the context of vaccination. Interestingly, HVTN505 conducted in the USA showed different FcγR SNPs associated with a different hazard ratio of HIV-1 acquisition from that of RV144. In the HVTN505 trial, patients receiving the vaccine had significantly higher incidences of HIV acquisition than those receiving placebo among participants carrying the FCGR2C-TATA haplotype or the FCGR3B-AGA haplotype. Moreover, an FCGR2A SNP (rs2165088) and two FCGR2B SNPs (rs6666965 and rs666561) influenced the correlation of anti-gp140 antibody-dependent cellular phagocytosis with HIV risk [81]. Of note, the HVTN505 and RV144 trials differed in a number of points, i.e., canarypox prime/protein boost in a general low-risk Thai population in RV144 versus DNA prime/rAd5 boost in a high-risk U.S. population of men who have sex with men in HVTN505.

These results indicate that the functional impact of a given FcγR polymorphism on the risk for HIV-1 acquisition is highly context specific, depending on the specific vaccine regimen but also on other factors, such as demographics, virus quasi-species, and genetic background [53, 81, 82].

## Discussion of future aspects

RV144 was the sole HIV-1 vaccine trial that showed a limited but statistically significant decreased infection risk [8, 10, 82]. As this protection was not associated with neutralization but with specific Ab types and Fc-mediated function, increased efforts were made to obtain a more in-depth characterization of the induced HIV-specific Ab response [10, 54, 82]. Indeed, in addition to HIV-specific Ab response and neutralizing activity, the specificity of the recognized epitope and Fc-mediated functions were investigated (Table 1). In addition, the FcR polymorphisms associated with infection outcome were explored [50–52, 54, 55, 81, 82]. However, taken individually, none of these factors could be associated with protection. For example, attempts to associate FcR genotypes with HIV outcome resulted in variable, sometime contradictory, results (Table 1). These results largely suggest that multiple Ab factors, including Ab class and subclass, structures, Fc domain interactions with Fc receptors, FcR locus copy number and FcR polymorphisms, may impact vaccine efficacy with synergistic or sometimes antagonistic effects [83]. Moreover, as Ab concentrations and FcR polymorphism frequencies vary according to ethnicities, analysis of correlates of infection risk need to take these additional parameters into consideration [63–65]. These results shed light on the complexity of the humoral response that may be correlated with a decreased risk of HIV-1 acquisition. Future vaccine strategies need to address humoral Ab induction as a whole challenging the different characteristics of the Abs and FcRs required to obtain the most promising combination of humoral responses associated with protection.
